# Draft Genome Sequence of Enterococcus mundtii SCPM-O-B-8398 (E28), Isolated from Fermented Milk in the Moscow Region, Russian Federation

**DOI:** 10.1128/MRA.00277-21

**Published:** 2021-07-01

**Authors:** Marat G. Teymurazov, Аlena А. Abaimova, Angelina A. Kislichkina, Olga I. Tazina, Vladimir V. Perelygin, Viktor D. Pokhilenko, Edward A. Svetoch, Nikolay N. Kartsev

**Affiliations:** a State Research Center for Applied Microbiology and Biotechnology, Obolensk, Russia; Loyola University Chicago

## Abstract

We report the draft genome sequence of the bacteriocin-producing Enterococcus mundtii strain SCPM-O-B-8398 (E28), which was isolated from fermented milk in the Moscow region, Russian Federation.

## ANNOUNCEMENT

Enterococcus mundtii is a Gram-positive, facultative, anaerobic, yellow-pigmented, nonmotile bacterium that occurs in natural environments, humans, and various animal species, as well as being detected in fermented dairy products due to the capability of lactic acid fermentation (1, 2). On the basis of homology in the 16S rRNA gene sequence, E. mundtii has been assigned as a member of the Enterococcus faecium group (3). This bacterium has low GC contents, ranging between 38% and 39%, and lacks catalase and cytochrome *c* oxidase enzymes (4).

This species has been studied in relation to the bacteriocins produced. Several strains of E. mundtii produce bacteriocins belonging to ribosomally synthesized peptide class IIa, which is characterized by low weight (4 to 6 kDa), heat stability, the presence of a consensus sequence of YGNGV in the N-terminal region, and high antilisterial activity (5). The most well-known and well-studied bacteriocins are mundticin ATO6, mundticin KS, and enterocin CRL35 (1, 5, 6).

E. mundtii SCPM-O-B-8398 (E28) is a bacteriocin-producing strain that was isolated from fermented milk that had been purchased from a retail outlet during microbiological screening in the Moscow region. Aliquots of milk were plated on petri dishes with nutrient medium number 1 (State Research Center for Applied Microbiology and Biotechnology [SRCAMB], Obolensk, Russia) and cultivated at 37°C for 24 h. The grown colonies were examined for antagonistic activity against Gram-positive and Gram-negative bacteria using the spot agar method. E. mundtii SCPM-O-B-8398 (E28) is active against the most tested strains responsible for spoiling meat, including Gram-positive strains of *Listeria* spp., *Enterococcus* spp., and Clostridium perfringens deposited in the SRCAMB microorganism collection.

The species identification of E. mundtii and detection of the mundticin KS gene were confirmed by PCR (7, 8). The stocks of strain E. mundtii were stored at −70°C in cryoprotective medium. Bacteria were grown at 37°C on nutrient medium number 1 (SRCAMB). DNA was isolated with the GenElute bacterial genomic DNA kit (Sigma-Aldrich, USA). Whole-genome sequencing was performed using an Illumina MiSeq instrument according to the manufacturer’s instructions. DNA libraries were prepared using the Nextera DNA library preparation kit. The MiSeq reagent kit v3 (300 cycles) was used for sequencing. A total of 371,178 paired-end reads (193,029,116 bases) were generated, and these yielded 102-fold coverage of the studied genome. The reads, without filtering, were assembled *de novo* using Unicycler v0.4.7 with default parameters (9). The draft genome of the strain is 3,389,182 bp in length and is composed of 263 contigs with a mean GC content of 38.2%. The *N*_50_ value is 59,895 bp. The final assembly was annotated with the NCBI Prokaryotic Genome Annotation Pipeline (PGAP) v4.11 (10).

Analysis of the draft genome of E. mundtii SCPM-O-B-8398 (E28) revealed the region that is homologous to the *mun* locus (encoding mundticin KS) of E. mundtii NFRI 7393 (GenBank accession number AB066267.1). The alignment was performed using Mauve (11) and BLAST (12). Default parameters were used for all software. Investigation of three genes included in the locus showed 100% nucleotide homology of the *munA* genes, encoding bacteriocin, and 99% homology of the *munB* and *munC* genes, encoding an ATP-dependent transporter and the mundticin KS immunity protein, respectively.

### Data availability.

The draft genome sequence has been deposited in the NCBI GenBank database under the accession number JABCAG000000000.1. Sequencing reads were deposited in the NCBI SRA database under the accession number SRR11550140.
